# Exploring the effect of changes to service provision on the use of unscheduled care in England: population surveys

**DOI:** 10.1186/1472-6963-7-61

**Published:** 2007-04-27

**Authors:** Alicia O'Cathain, Emma Knowles, James Munro, Jon Nicholl

**Affiliations:** 1 Medical Care Research Unit, School of Health and Related Research, University of Sheffield, Regent Court, Sheffield S1 4DA, UK

## Abstract

**Background:**

Unscheduled care is defined here as when someone seeks treatment or advice for a health problem without arranging to do so more than a day in advance. Recent health policy initiatives in England have focused on introducing new services such as NHS Direct and walk in centres into the unscheduled care system. This study used population surveys to explore the effect of these new services on the use of traditional providers of unscheduled care, and to improve understanding of help seeking behaviour within the system of unscheduled care.

**Methods:**

Cross-sectional population postal surveys were undertaken annually over the five year period 1998 to 2002 in two geographical areas in England. Each year questionnaires were sent to 5000 members of the general population in each area.

**Results:**

The response rate was 69% (33,602/48,883). Over the five year period 16% (5223/33602) 95%CI (15.9 to 16.1) of respondents had an unscheduled episode in the previous four weeks and this remained stable over time (p = 0.170). There was an increased use of telephone help lines over the five years, reflecting the change in service provision (p = 0.008). However, there was no change in use of traditional services over this time period. Respondents were most likely to seek help from general practitioners (GPs), family and friends, and pharmacists, used by 9.0%, 7.2% and 6.3% respectively of the 5815 respondents in 2002. Most episodes involved contact with a single service only: 7.0% (2363/33,602) of the population had one contact and 2% (662/33602) had three or more contacts per episode. GPs were the most frequent point of first contact with services.

**Conclusion:**

Introducing new services to the provision of unscheduled care did not affect the use of traditional services. A large majority of the population continued to turn to their GP for unscheduled health care.

## Background

Much health care use in the United Kingdom (UK) is provided at less than 24 hours notice. This can be termed 'unplanned', 'unscheduled' or 'urgent' care. There are numerous health services which people can access for unscheduled care in the UK, in particular general practice, the emergency ambulance service, and accident and emergency departments. Concerns have been expressed about the ability of these traditional health services to deal with rising demand for unscheduled care [1]. Recent UK health policy initiatives have focused on introducing new services into the system of unscheduled care, either to provide care or to guide patients to the most appropriate traditional service. In particular NHS Direct is a 24 hour telephone assessment service offering self care advice and direction of callers to other services, and was introduced in 1998 with expansion to the whole of England in 2000; walk in centres offer treatment and advice from nurses without the need for an appointment, and were introduced in 19 geographical areas in England in 1999 with expansion to 82 centres by 2004. The expectation of policy makers was that these new services would reduce demand for traditional services [2], by facilitating self care, directing people to more appropriate services, and in the case of walk in centres by offering treatment. These changes to service provision even led to the suggestion that general practitioners could no longer claim to be the gatekeepers of the National Health Service (NHS) [3].

Little is known about how people use unscheduled care because research has tended to focus on general use of health services [4] rather than considering scheduled and unscheduled care separately, or research has focused only on unscheduled care used outside normal working hours, [5] when it is used both in and out of hours. The increasing number and type of services offering unscheduled care has led policy makers and researchers to consider the services offering unscheduled care as a system [6]. Routinely available data has been used to explore the dynamics of this system, with the limitation that data are only available for some parts of the system [6]. There has also been an emphasis on health services when informal and self care is a hidden part of the supply of health care which can act as both an alternative and a supplement to formally provided care [7]. A survey of the general population offers an alternative approach to exploring the use of unscheduled care which allows for the study of a wide range of services and informal care.

The aims of this study were to use population surveys to explore the effect of changes to the provision of unscheduled care on the use of a range of traditional services, and to explore the use of unscheduled care to increase understanding of this issue.

## Methods

We undertook population surveys in the three geographical areas where NHS Direct was first introduced in 1998. These areas included a town in the south of England, a mixed urban and rural area in north west England, and a city and rural area in north east England. A walk in centre opened in the third area in 1999/2000. We undertook the first survey in 1998, immediately before the introduction of NHS Direct, and repeated it annually for the five year period up to 2002. In each year, we selected a random sample of 5,000 individuals (of all ages) from the NHS register in each area. In the city in the north east, the health authority would not provide a population sample, so we selected 3,000 names randomly from the local electoral roll (which includes only those aged 18 and over) and added these to the sample of 2,000 provided by the health authority for the adjacent rural area. The health authority covering the city in the south of England provided a population sample for the first three years only and therefore was excluded from the analysis. The local health authority, or the research team for the electoral roll sample, posted a questionnaire and covering letter, with up to two reminders to non-respondents at fortnightly intervals. The intention was to post the survey in February each year but because of difficulties in obtaining samples this occurred up to June in some years. Guardians and parents were asked to complete questionnaires on behalf of children.

The survey was described as a 'health care survey for the NHS' and remained unchanged throughout the study with the exception of the last page which covered different issues each year. Unscheduled care is defined here as when someone seeks treatment or advice for a health problem without arranging to do so more than a day in advance. Respondents were asked whether they had sought help or advice for a health problem in the previous four weeks, however minor (an 'episode'). Further details were sought for the most recent unscheduled episode. A list of people and services was provided and respondents were asked to report which ones they had contacted. Family and friends were included in this list to gain an understanding of use of informal care. Generic descriptions of services were given such as 'a family doctor (GP) from my usual practice', 'a family doctor (GP) not from my usual practice', 'a hospital accident and emergency department'. NHS Direct was not named because it did not exist in 1998; instead the category 'telephone help line' was used. An open option of 'someone else' was included where respondents could write the names of other professionals or services used; contacts with 'walk in centres' were captured here. Respondents were asked to indicate the order in which they sought help from any services contacted. Age and gender were collected each year, and in 2002 socio-economic variables were also collected. Approval was given by Trent Multi-centre Research Ethics Committee.

### Analysis

We used SPSS to analyse the structured data. A researcher (EK) read and coded responses to the unstructured option of the question about type of service or person contacted; when respondents used the specific name of a service in their locality we identified the service and allocated it to a generic type. We undertook logistic regression to examine changes over time in the use of different services for unscheduled care. The dependent variable was whether or not members of the general population had contacted a specific person or service for unscheduled care in the previous four weeks. We adjusted for age and gender of respondents, month of response, and geographical area. We adjusted for the month in which the questionnaire was completed by each respondent because the incidence and type of health problems are likely to vary seasonally. Because of the large number of tests undertaken, we used a p-value of 0.01 to indicate statistically significant change.

## Results

### Response rates and description of respondents

The response rate overall was 69% (33,602/48,883) after 'return to senders' were removed from the denominator. Response rates were high in each year of the survey, at just above 70%, which is excellent for a general population survey (Table 1). The exception was 2002 when the response rate fell to 60%. In this year socio-economic questions were included in the questionnaire. These may have been perceived as sensitive questions by potential respondents, causing an adverse effect on the response rate [8]. The characteristics of respondents are shown in Table 1. Over time we noted a decreasing proportion of young adult respondents (18–34 years olds) and an increasing proportion of 35–64 years olds. All analyses of change over time have been adjusted for age and sex.

**Table 1:** Response rates and description of respondents to the population surveys by year

	**1998**		**1999**		**2000**		**2001**		**2002**		**Total**		**Census**
	**%**	**n**	**%**	**n**	**%**	**n**	**%**	**n**	**%**	**n**	**%**	**n**	**2001 %**
Responses	74	(7217)	71	(6907)	70	(6777)	70	(6886)	60	(5815)	69	(33,602)	
Sex													
Male	47	(3370)	45	(3095)	46	(3081)	45	(3089)	44	(2552)	45	(15,187)	48
Female	53	(3834)	55	(3800)	54	(3676)	55	(3774)	56	(3241)	55	(18,325)	52
Age													
0–4	4	(284)	4	(243)	4	(240)	4	(253)	4	(207)	4	(1227)	6
5–17	13	(905)	12	(808)	13	(882)	13	(877)	13	(748)	13	(4220)	16
18–34	22	(1566)	21	(1456)	19	(1311)	19	(1271)	17	(970)	20	(6574)	} 57
35–64	42	(3036)	44	(3019)	44	(2948)	46	(3117)	46	(2669)	44	(14,789)	}
65+	20	(1401)	20	(1366)	20	(1363)	20	(1339)	21	(1185)	20	(6654)	20
Home ownership													
Yes	-	-	-	-	-	-	-	-	79	(4494)	79	(4494)	66
No									21	(1229)	21	(1229)	34
Car ownership													
None	-	-	-	-	-	-	-	-	20	(1166)	20	(1166)	40
One									44	(2528)	44	(2528)	42
Two or more									37	(2120)	37	(2120)	18

### Changes in the use of different services for unscheduled care

Taking all years together 37% (12277/33602, 95%CI 36.7, 37.3) of respondents reported that they had sought treatment or advice – scheduled or unscheduled – for any health problem, however minor, in the previous four weeks. 16% (5223/33602, 95% CI 19.1, 16.1) of respondents reported an episode of unscheduled care (Table 2). There was no evidence that these proportions changed over time for seeking any type of care (p = 0.519) or for seeking unscheduled care (p = 0.170). After adjustments, a statistically significant change was found only in the use of telephone help lines, which was likely to be the increasing use of NHS Direct over this time period. There was no indication of change in use of traditional services or informal care over time, although there was a possible reduction in use of dentists.

**Table 2:** Changes in the use of different sources of unscheduled care in the previous four weeks (N = 33602 respondents)

Service*	1998–2002 % (n)	1998 % (n)	2002 % (n)	Adjusted odds ratios**					P-value***
				1998	1999	2000	2001	2002	
Unscheduled care	15.5 (5223)	16.0 (1155)	15.3 (887)	1	1.06	0.95	0.99	1.05	0.170
Usual GP	9.7 (3245)	10.5 (758)	9.0 (521)	1	0.97	0.92	0.93	0.92	0.763
Family/friends	7.7 (2582)	7.7 (558)	7.2 (421)	1	1.12	1.02	0.99	1.00	0.341
Pharmacist	6.9 (2309)	7.5 (541)	6.3 (368)	1	1.02	0.91	0.92	0.99	0.373
A&E/999 ambulance	1.8 (605)	1.5 (105)	2.1 (120)	1	1.32	1.19	1.12	1.11	0.336
Telephone helpline	1.0 (339)	0.7 (47)	1.5 (88)	1	1.39	1.51	1.78	2.47	0.008
Someone else at GP practice but not a doctor	1.2 (417)	1.2 (89)	1.5 (87)	1	1.02	0.84	0.98	1.27	0.223
Outpatient clinic	1.2 (402)	1.1 (77)	1.3 (74)	1	1.23	1.26	0.85	1.06	0.112
GP, not usual GP	0.9 (296)	0.9 (66)	0.9 (51)	1	1.11	0.76	0.97	1.03	0.380
Hospital admission	0.7 (227)	0.6 (42)	0.8 (49)	1	1.45	1.07	0.94	1.20	0.260
Dentist	0.7 (250)	1.0 (70)	0.7 (42)	1	0.85	0.58	0.59	0.78	0.044
Complementary therapist	0.5 (157)	0.5 (35)	0.4 (22)	1	0.74	1.34	1.09	0.96	0.236
Physiotherapist	0.4 (148)	0.4 (32)	0.4 (21)	1	1.02	0.92	0.90	0.53	0.460
Walk in centre	0.1 (49)	0	0.3 (20)	-	-	1	1.78	1.80	0.293

*ordered in descending order of number of contacts in population in 2002, only contacts of 20 or more in 2002 reported

** adjusted for age, sex, area, and month of response

*** for change over time in odds ratios between 1998 and 2002

People may contact a number of services in any episode of unscheduled care. Given that a role of NHS Direct was to direct people to appropriate services, it is most likely that this new service affected the first contact that people made. Formal care only was studied in terms of which service was contacted first, how many services were contacted in each episode, and the most common pathways taken through services by the general population. 89% (4665/5223) of respondents who had had an unscheduled episode provided data on the number and order of contacts with services for their most recent unscheduled episode. The increasing use of telephone help lines for first contact was evident but there was no indication of a change in use of traditional services for the first contact (Table 3). Using multinomial regression, with adjustments for potential confounders, the numbers of contacts per episode made in the population did not change over time (p = 0.201).

**Table 3:** First contact services^+^ for unscheduled care in previous four weeks (N = 33602 respondents)

Service	1998–2002 % (n)	1998 % (n)	2002 % (n)	Adjusted odds ratios					P-value*
				1998	1999	2000	2001	2002	
GP	8.1 (2690)	8.9 (632)	7.5 (427)	1	0.98	0.95	0.95	0.95	0.931
Pharmacist	2.2 (718)	2.3 (162)	2.0 (117)	1	1.15	0.88	1.03	1.13	0.182
A&E/999 ambulance	1.0 (323)	0.9 (67)	0.9 (54)	1	1.18	1.08	0.83	0.85	0.410
Telephone helpline	0.5 (176)	0.2 (16)	0.9 (49)	1	2.30	2.75	3.82	4.85	0.001
Practice staff	0.6 (195)	0.5 (36)	0.8 (45)	1	1.27	0.79	1.19	1.54	0.112
Dentist	0.5 (167)	0.7 (47)	0.5 (27)	1	0.79	0.59	0.67	0.82	0.288
Other	1.2 (385)	1.2 (82)	1.2 (70)	1	0.90	0.99	1.02	0.97	0.947

* for change over time in odds ratios between 1998 and 2002

+ family and friends not included

### Exploring the use of unscheduled care

As reported above, 16% of respondents reported using unscheduled care in the previous four weeks. This proportion was not consistent across sub-groups of the population (Table 4). Children under 5 years old were twice as likely to seek unscheduled care as other age groups, women were more likely to seek unscheduled care than men, and people who did not own their own homes were more likely to seek unscheduled care than home owners. There was no difference in help-seeking behaviour by car ownership.

**Table 4:** Proportion of respondents seeking unscheduled care in the previous four weeks, by age, sex, and socio-economic status 1998–2002

Characteristic of respondents	%	n	N	p-value
Age				0.001
0–4	32	388	1227	
5–17	18	764	4220	
18–34	17	1099	6574	
35–64	14	2011	14789	
65+	14	941	6654	
				
Sex				0.001
Male	13	2041	15187	
Female	17	3174	18325	
				
Home				0.001
Owner	14	648	4494	
Not	18	225	1229	
				
Car				0.302
None	14	165	1166	
One	16	405	2528	
Two+	15	317	2120	

Total	16	5223	33602	

GPs and pharmacists were the commonest sources of help and advice for the most recent unscheduled episode (Table 2). People also made extensive use of informal care from family and friends. A considerable amount of unscheduled care took place in hours as well as out of hours: 49% (2572/5223) of unscheduled episodes occurred in hours, 40% (2086/5223) out of hours, and 11% (565/5223) of respondents did not give a time and day at which help was sought. As reported above, respondents were asked to give further details about contacts with services only. By far the most common first contact was the GP (Table 3). In 2002, when new services had been established for at least 3 years, the five main services contacted first in an episode of unscheduled care were the GP, the pharmacy, emergency care, telephone help lines, and general practice staff (Table 3).

Respondents reported between one and ten contacts with services, although a large majority reported three or fewer. Most episodes involved contact with a single service only: 7.0% (2363/33,602) of the population had one contact, 4.8% (1629/33,602) had two contacts, and 2% (662/33602) had three or more contacts per episode. Over the five year period, of those who had an episode of unscheduled care and reported the number of contacts with services, 51% (2363/4654) had one contact only, 35% (1629/4654) had two contacts, and 14% (662/4654) had three or more contacts during the episode. In 2002, when new services had been established for three years, by far the most common pathway of service use for unscheduled care was one contact with a GP (Table 5). It is interesting to note that 'pharmacy only' and 'help line only' were common pathways, indicating the frequency with which the population dealt with minor illness without recourse to traditionally overloaded services. Telephone help lines featured at the start of a number of common pathways.

**Table 5:** Commonest pathways for service use for unscheduled care in previous four weeks (N = 5723 respondents in 2002)*

Contact 1	Contact 2	Contact 3	Number on pathway	% of N = 5723 respondents
GP			225	3.9
GP	Pharmacy		85	1.5
Pharmacy			65	1.1
GP	Other		39	0.7
A&E/999/ambulance			33	0.6
Other			29	0.5
Pharmacy	GP		22	0.4
Practice staff			20	0.3
Dentist			18	0.3
Other	GP		13	0.2
Helpline			10	0.2
Pharmacy	GP	Pharmacy	10	0.2
GP	Other	Other	9	0.2
Practice staff	Pharmacy		9	0.2
GP	GP		8	0.1
GP	Practice staff		8	0.1
Helpline	A&E/999/ambulance		7	0.1
Helpline	GP		6	0.1
GP	A&E/999/ambulance		6	0.1
Helpline	GP	Pharmacy	6	0.1
A&E/999/ambulance	Other		5	0.1
Other	Other		5	0.1

* 5723 of 5815 respondents in 2002 gave details about the number and type of contacts made with services

## Discussion

The distribution of use of unscheduled care and first contact with a service remained relatively stable over time despite the addition of new developments in unscheduled care provision, particularly NHS Direct and walk in centres. Expected reductions in the use of traditional services were not apparent. A study of the impact of NHS Direct on other services, using routine data, found no effect on ambulance services or accident and emergency, and a small effect on out of hours general practice services [9] which persisted in the longer term [10]. Similarly, walk in centres have been observed to be associated with a small but non-statistically significant reduction in consultations at accident and emergency departments and general practices close to the walk in centres [11], and no change in the daily rate of emergency GP consultations and daily rate of attendances at out of hours services [12]. Taken together, these findings add some weight to the assertion that new developments in the provision of formal health services for unscheduled care have been associated with little or no measurable change in the overall volume of use of other NHS services [13]. This may be explained by the length of time it takes for new services to become established or by the 'low dose' of new services in a large and complex system – only 6% (49/789) of all first contacts with services for unscheduled care in 2002 were with telephone help lines. Having said this, telephone help lines – likely to be NHS Direct – had become one of the top five providers of formal unscheduled care in England and one of the main five first contact services for unscheduled care.

The proportion of people with an unscheduled care episode in the previous four weeks was estimated to be 16%. The relatively stable rate for contacting services for unscheduled care over time appears to contradict reports of increasing use of services in England. For example the proportion of adults and children who consulted a general practitioner in the previous 14 days increased in Britain from 12% in 1972 to 15% in 2002, with a peak in the mid 1990s [14]. However, the period between 1998 and 2002 was relatively stable compared with earlier time periods. Indeed a study of the emergency care system using routine data showed patient contacts did not increase for traditional services between 1998 and 2001 [6].

The general population were most likely to turn to general practitioners, pharmacists, and family and friends for unscheduled care and make first contact with a service via GP, pharmacy and emergency services. The dominant role of the general practitioner in the provision of unscheduled care has been shown previously for out of hours services, where 45% of patient contacts were with general practitioners [5]. In another study where unscheduled care was measured in 1996, people were over four times more likely to seek help from a general practitioner than accident and emergency services, a similar ratio to the one found here [15]. Concerns about the loss of the gatekeeper role of the GP in the light of changes to the system of unscheduled care do not seem justified [3].

### Strengths and limitations of this study

Response rates to the surveys were good. No information was available about non-responders, but given that the salience of the topic increases survey response rates [8], people who used services in the previous four weeks were probably more likely to have responded, so it is probable that point estimates of service use are higher among the respondents than in the population at large. Census data for the local authority areas most closely matched to the populations included here showed that the survey respondents were less likely to be male, less likely to be children and young adults, more likely to be middle aged adults, more likely to be home owners and more likely to own two or more cars. It is unclear from this whether we have under or over estimated the use of unscheduled care but it is likely that the accuracy of the estimate is not as good as implied by the 95% confidence interval.

Generic terms for services were used on the questionnaire, such as 'telephone help line' rather than NHS Direct, and use of walk in centres was collected by respondents writing down which 'other service' they had used. This may have affected percentages of people estimated to have contacted these services. Service provision differs in areas of England, and this is highlighted by the fact that only one area of the two areas included here had a walk in centre available. The geographical areas were not selected to be representative of England and in fact both areas were in the north of the country. However, they are standard populations covering a mixture of urban and rural areas and so the findings are likely to be generalisable to England. The findings may not be transferable to other health care systems.

A key limitation was the lack of control areas where new services were not introduced in this time period. Population surveys of this size are resource intensive and we did not have the resources to extend the survey into control areas. It was also the case that new services were developing at a rapid rate and there was a risk of establishing control areas which would quickly change status. NHS Direct became nationwide in 2000 and further waves of walk in centres were introduced over the time period of this study. It is also the case that changes have continued to occur in the formal provision of services for unscheduled care since 2002. Contacts with NHS Direct have increased from around 6 million calls in 2002 to 7 million calls in 2005 (House of Commons Hansard Written Answers) and changes have occurred to the role of GPs in the provision of out of hours care. Nonetheless the period studied here was one of considerable change, and yet GPs remained a key source of formal unscheduled care.

## Conclusion

Recent changes to the provision of unscheduled care did not affect traditional providers of this care because, although this new service provision dealt with large numbers of people, the contribution was essentially 'low dose' in a large and complex system. General practitioners, pharmacists, and family and friends are key providers of unscheduled care. The formal provision of unscheduled care is dominated by general practice and this has remained the case even with the recent introduction of new services into the health care system. Patients can take a variety of routes into, and through, services providing unscheduled care. In the future it will be important to explore patient satisfaction, patient outcomes, and impact on other parts of the health care system associated with these different routes through the system of unscheduled care.

## Competing interests

The authors declare that they have no competing interests.

## Authors' contributions

AOC analysed and interpreted the data, and wrote the paper. EK collected the data, and contributed to analysing and interpreting the data and writing the paper. JM and JPN conceived and designed the study, and contributed to analysing and interpreting the data and writing the paper. All authors read and approved the final manuscript. AOC acts as guarantor.

## Pre-publication history

The pre-publication history for this paper can be accessed here:


